# Multi‐Parametric MRI Approach at 3 T and 7 T for Assessing Skeletal Muscle Pathology in Myofibrillar Myopathies: A Pilot Study

**DOI:** 10.1002/jcsm.70245

**Published:** 2026-03-19

**Authors:** Claudius S. Mathy, Lena V. Gast, Christian Holtzhausen, Teresa Gerhalter, Christoph Stuprich, Matthias Türk, Rafael Heiss, Benjamin Marty, Frederik B. Laun, Julia V. Wanschitz, Simon Hametner, Arnd Dörfler, Michael Uder, Tobias Bäuerle, Armin M. Nagel, Rolf Schröder

**Affiliations:** ^1^ Institute of Radiology Universitätsklinikum Erlangen, Friedrich‐Alexander‐Universität Erlangen‐Nürnberg (FAU) Erlangen Germany; ^2^ Institute of Neuropathology Universitätsklinikum Erlangen, Friedrich‐Alexander‐Universität Erlangen‐Nürnberg (FAU) Erlangen Germany; ^3^ Department of Neurology Medical University of Graz Graz Austria; ^4^ Department of Neurology Universitätsklinikum Erlangen, Friedrich‐Alexander‐Universität Erlangen‐Nürnberg (FAU) Erlangen Germany; ^5^ Centre for Rare Diseases Erlangen (ZSEER), Universitätsklinikum Erlangen, Friedrich‐Alexander‐Universität Erlangen‐Nürnberg (FAU) Erlangen Germany; ^6^ NMR Laboratory Institute of Myology Paris France; ^7^ Department of Neurology Medical University of Innsbruck Innsbruck Austria; ^8^ Division of Neuropathology and Neurochemistry, Department of Neurology Medical University of Vienna Vienna Germany; ^9^ Department of Neuroradiology Universitätsklinikum, Friedrich‐Alexander‐Universität Erlangen‐Nürnberg (FAU) Erlangen Germany; ^10^ Department of Diagnostic and Interventional Radiology University Medical Center of Johannes Gutenberg‐University Mainz Germany; ^11^ Division of Medical Physics in Radiology German Cancer Research Center (DKFZ) Heidelberg Germany

**Keywords:** distal myopathy, myofibrillar myopathy, quantitative MRI, sodium (^23^Na) and potassium (^39^K) MRI, ultra‐high field MRI

## Abstract

**Background:**

Myofibrillar myopathies (MFM) form a large group of clinically and genetically heterogeneous protein aggregate diseases. We investigated whether a novel quantitative MRI protocol can reveal new aspects of structural and biochemical muscle pathology in three classic MFM subtypes.

**Methods:**

MRI of the lower legs was performed in nine MFM patients with filamin‐C (*FLNC*; *n* = 5), desmin (*DES*, *n* = 2) and LIM domain binding 3 (*LDB3*; *n* = 2) gene mutations, one patient with non‐MFM, filamin‐C related distal myopathy (4 males, 6 females, 51.0 ± 7.7 years) and 10 age‐matched healthy control subjects (5 males, 5 females, 50.0 ± 11.0 years). ^1^H MRI at 3 T addressed fatty replacement and edema‐like changes as well as quantitative measurements of proton density fat fraction (PDFF) and water T_2_ relaxation times. ^39^K/^23^Na MRI at 7 T was employed to determine apparent tissue potassium and tissue sodium concentrations (aTPC/aTSC).

**Results:**

T_1_‐weighted and T_2_‐weighted STIR imaging showed the highest degree of fat replacement in the soleus and gastrocnemius medialis muscle regions and the highest degree of edema‐like changes in the extensor regions in all 10 myopathy patients. The lowest degree of fat replacement and edema‐like changes was present in the gastrocnemius lateralis muscles. Marked fatty replacement of peroneus muscles was also present in DES‐related MFM and *FLNC*‐related distal myopathy. Muscular PDFF values were significantly increased in all MFM patients (*p* = 0.003 ‐ < 0.001) with 60 and 35 of 63 muscles analysed showing increased mean PDFF (> 10% and > 50%). When excluding the muscles with PDFF > 50%, the median water T_2_ was significantly increased in all muscle regions of MFM patients with the exception of the tibialis anterior and posterior muscles. Fat‐corrected aTSC values in MFM patients were significantly increased compared to healthy controls (55.6 ± 16.3 mM vs. 23.2 ± 5.5 mM, *p* < 0.001) in all muscles but peroneus muscles, whereas fat‐corrected aTPC values were reduced in all muscles except for gastrocnemius lateralis, tibialis posterior and peroneus muscles (75.4 ± 13.3 mM vs. 108.9 ± 9.9 mM, *p* < 0.001).

**Conclusions:**

Quantitative PDFF measurements and water T_2_ mapping serve as valuable tools to objectively quantify fat and edema‐like changes in MFM. Furthermore, changes in potassium/sodium ion balance in the lower leg muscles of MFM patients could serve as new markers to quantify the extent of biochemical changes in individual muscle regions. Further longitudinal evaluation is required to validate whether they are sensitive to changes prior to a high degree of fat replacement.

## 1 Introduction

Myofibrillar myopathies (MFM) encompass a large group of sporadic and familial neuromuscular conditions of considerable clinical and genetic heterogeneity [1, 2, 3]. The shared morphological hallmark of the skeletal muscle pathology in all MFM subforms—irrespective of the individual gene defect—is the presence of desmin‐positive sarcoplasmic protein aggregates in conjunction with degenerative alterations affecting the structural and functional organization of the myofibrillar apparatus [4]. Over the last 30 years, an increasingly long and complex list of human gene defects has been identified which cause MFM subtypes with autosomal‐dominant, autosomal‐recessive and X‐linked modes of inheritance [1]. While the vast majority of MFM are caused by mutations in genes coding for sarcomeric (filamin‐C (*FLNC*), LIM domain binding 3 (*LDB3*), *MYOT*, *BAG‐3* and *TTN FHL1*) and extrasarcomeric (desmin (*DES*), *PLEC* and *SVIL*) cytoskeletal proteins, less frequently encountered defects in genes coding for proteins with essential functions in protein quality control (*CRYAB*, *DNAJB6*, *HSPB8*, *KLCH24* and *VCP*) add to the complexity of MFM genetics [1, 2, 3]. To date, no specific treatment is available for this group of neuromuscular disorders. Previous imaging studies with visual assessment of patterns of adipose tissue and edema‐like changes using T_1_‐ and T_2_‐weighted short‐tau‐inversion‐recovery (STIR) ^1^H MRI have found quite characteristic patterns of skeletal muscle involvement in different MFM subtypes at the level of the pelvis, thigh and lower leg [5, 6]. However, MRI studies that can quantify and monitor the individual disease pattern and progression as well as the effects of therapeutic interventions in MFM are lacking.

To assess the skeletal muscle pathology in MFM, we performed a novel multi‐parametric approach of ^1^H MRI performed at 3 T and ^23^Na/^39^K MRI performed at 7 T in the lower legs of nine MFM patients caused by FLNC, DES and LDB3 gene mutations, one patient with non‐MFM, FLNC‐related distal myopathy and 10 age‐matched healthy control subjects.

## 2 Materials and Methods

### 2.1 Study Population

Nine patients with genetically confirmed myofibrillar myopathies due to heterozygous *FLNC* (*n* = 5), *DES* (*n* = 2) and *LDB3* (*n* = 2) mutations and one patient (P6) with FLNC‐related distal myopathy as a non‐MFM disease control (4 males, 6 females, mean age = 51.0 ± 7.7 years, range 36–61 years) were prospectively enrolled in this study. Previous myopathological work‐up of a diagnostic skeletal muscle biopsy from patient P6 showed a myopathic pattern but no protein aggregation pathology. In addition, 10 age‐matched healthy controls (5 males, 5 females, mean age 50.0 ± 11.0 years, range 32–64 years) without any history of neuromuscular pathologies and no current acute injuries of the lower limbs were recruited. All study participants were asked to forego sports 48 h before MRI examinations to reduce the impact of muscle soreness on quantitative MRI measures. The study was approved by the local institutional ethics committee (No. 254_21B) and written informed consent was obtained from all participants.

### 2.2 Laboratory Investigation

Serum levels of potassium, sodium and creatine kinase (CK) were determined in all participants imminent to individual ^1^H MRI examinations.

### 2.3 MRI Examinations

^1^H MRI examinations were performed at a 3 T whole‐body MR system (Magnetom Vida, Siemens Healthineers AG, Erlangen, Germany) using an 18‐channel flexible RF coil in combination with the spine coil integrated into the scanner table. Both lower legs were positioned side by side on two holders shaped like the volume RF coil used for ^23^Na/^39^K MRI at 7 T, to facilitate image co‐registration. The largest circumference of each calf was marked to ensure the same positioning during ^23^Na/^39^K MRI, which was performed subsequently to ^1^H MRI. The ^1^H RF coil was placed on top of the legs.

First, T_1_ weighted turbo spin echo (TSE) as well as T_2_ weighted STIR TSE images of both lower legs were acquired to qualitatively assess the fatty replacement and edematous/inflammatory processes within muscle tissue (acquisition parameters summarized in Table S1). Subsequently, the less affected leg was selected based on the T_1_ weighted image to optimize the proportion of more intact muscle tissue for quantitative MRI acquisitions. A 6‐point Dixon‐type acquisition with monopolar readout was performed to determine the muscular proton‐density fat fraction (PDFF). In addition, a multi‐echo spin‐echo (MESE) sequence was acquired for water T_2_ determination. All ^1^H MR images were acquired in axial slice direction.

^23^Na and ^39^K MRI measurements were conducted using a whole‐body 7 T MRI system (Magnetom Terra. X, Siemens Healthineers AG, Erlangen, Germany) using a dual‐tuned ^23^Na/^39^K quadrature birdcage calf RF coil (Rapid Biomedical GmbH, Rimpar, Germany). Both MR scanners (3 T and 7 T) were located in adjacent rooms. During ^23^Na/^39^K MRI, the leg was placed on top of a reference phantom holder containing five compartments filled with NaCl and KCl solutions ([Na^+^]/[K^+^] = 10/240, 20/210, 25/180, 30/150 and 40/120 mM), which were used for quantitative evaluation of the ^23^Na/^39^K MRI data. B_0_ shimming was performed based on the ^23^Na MR signal using a constrained regularized algorithm [7]. Quantitative ^39^K and ^23^Na images were acquired using a 3D acquisition‐weighted Stack‐of‐Stars (AW‐SOSt) sequence applying a non‐selective excitation pulse (see Table S1) [8]. Furthermore, a ^23^Na inversion recovery (IR) MRI sequence applying a 180° inversion pulse to suppress ^23^Na signal from fluid compartments was acquired. This ^23^Na IR approach was shown to provide a weighting towards changes in the intracellular sodium concentrations [9].

### 2.4 MRI Data Evaluation

Semi‐quantitative analysis of the T_1_‐weighted and T_2_‐weighted STIR images was performed as described previously [10, 11]. For fat replacement/edema on T_1_‐weighted/T_2_‐weigthed STIR, a four‐point visual scale was used by C.S.M. with 3 years under supervision of R.H. with 10 years of experience in musculoskeletal MRI: Grade 1: homogeneous hypointensity (normal muscle), Grade 2: slight hyperintensity with patchy intramuscular signal intensity (SI) changes (< 50% of muscle cross‐section), Grade 3: marked hyperintensity with patchy but widespread intramuscular SI changes (> 50% of muscle cross‐section) and Grade 4: homogeneous hyperintensity in whole muscle. For quantitative evaluation of the MRI data, T_1_‐weighted ^1^H images were segmented into gastrocnemius medialis/lateralis (GM/GL), soleus (SOL), tibialis anterior/posterior (TA/TP), peroneus (PER) and extensor longus digitorum (EDL) muscles using a semi‐automatic image segmentation tool (Dafne, example see Figure S1) [12]. In addition, the subcutaneous fat tissue and the whole muscle tissue were segmented manually. Segmentation masks were co‐registered/transformed to all quantitative MRI acquisitions including the ^23^Na and ^39^K MRI data using non‐rigid image registration using Elastix [13].

Separation of the water and fat signals was achieved based on the complex MR signal of the 6‐pt Dixon‐type acquisition using a GraphCut algorithm implemented in Matlab, assuming an 8‐peak fat model and single *R*_2_* relaxation rate [14]. PDFF maps were subsequently calculated based on the separated water and fat images and average PDFF values for the individual muscle compartments were determined based on the segmentation masks.

Water T_2_ times were determined based on the MESE data using a tri‐exponential fit model implemented in Python to account for increased T_2_ relaxation times of fat compared to muscle tissue [15]. As this fit approach is not suited for very high fat fractions, muscles with a mean PDFF above 50% were excluded from the quantitative evaluation of water T_2_.

The reconstruction and post‐processing of the ^23^Na and ^39^K MRI data was performed offline using custom‐written Matlab tools as described in detail in Gast et al. [16]. Partial volume effects due to the low spatial resolutions of the ^23^Na and ^39^K MRI data were corrected using a region‐based partial volume correction (PVC) approach based on the segmentation masks for individual tissue regions [17, 18]. The PVC yielded average corrected ^23^Na and ^39^K signal intensities for each compartment, which were additionally corrected for relaxation weighting due to different relaxation properties of muscle/fat tissue and reference solutions. Quantification of apparent tissue sodium (aTSC) and potassium (aTPC) concentration values was then performed based on the partial volume and relaxation corrected signal intensities by a linear regression assuming known concentrations for the five external reference compartments containing NaCl and KCl solution. As sodium and potassium concentrations in fatty tissue are reduced compared to muscle tissue (aTSC_fat_ = 7.9 mM [19], aTPC_fat_ ≈ 0 mM), the aTSC and aTPC values were further corrected taking into account the measured average PDFF values of the individual muscles [19]. Similarly, the mean ^23^Na IR SI was determined in fully fat‐replaced muscles of MFM patients (^23^Na IR signal_fat_ = 2.5 a.u.) and used to determine ^23^Na IR signal intensities in remaining muscle tissue. To avoid inaccuracies due to low ^23^Na and especially ^39^K SI in strongly fat‐replaced muscles, muscle regions with a PDFF > 50% were also excluded from the evaluation of fat‐corrected tissue ion concentrations, as was the case with quantitative ^1^H MRI values. Relative ^23^Na signal intensities were calculated with reference to aTSC. Therefore, a linear regression between fat‐corrected ^23^Na IR signal intensities and aTSC was first determined based on all muscle regions of the controls and all values that conformed to the linear regression were set to 1.

### 2.5 Statistical Analysis

Results are presented as median, mean ± SD. Statistical analysis was performed using the MATLAB statistics toolbox (Matlab 2019b, The MathWorks). Values were checked for normality using the Lilliefors test. In case normality was indicated, individual muscle compartments were evaluated by unpaired *t*‐tests and Pearson correlations between different variables were calculated. In the opposite case, non‐parametric methods were used, including Mann–Whitney *U* tests and Spearman's correlations. A *p*‐value < 0.05 was considered significant. Muscle specific *p‐*values were adjusted for multiple testing using the Dubey–Armitage–Parmar procedure [20], which takes intermuscular dependence into account by calculating intermuscular correlations. In the extreme cases of no correlation (*r* = 0), this results in the same correction as the conservative Bonferroni procedure; in the case of complete correlation (*r* = 1), the corrected values remain the same as the uncorrected original values. For comparison, unadjusted and adjusted *p*‐values for muscular‐specific testing for all quantitative parameters are presented in Table S2. Effect sizes were included by calculating Pearson correlation coefficients r_eff_. All statistical analyses and boxplot presentations contain only data from the MFM patients; Patient P6 with non‐MFM, FLNC‐related distal myopathy was always considered separately.

## 3 Results

### 3.1 Clinical, Laboratory and Genetic Findings

The study cohort consisted of nine patients suffering from MFM, one patient with non‐MFM distal myopathy (4 males, 6 females, mean age = 51.0 ± 7.7 years, range 36–61 years) and 10 age‐matched healthy control subjects (5 males, 5 females, mean age 50.0 ± 11.0 years, range 32–64 years). Detailed information on age, sex, disease onset and duration, clinical phenotype, CK levels, individual gene defect, mobility score, muscle strength (MRC grades) and atrophy of the 10 myopathy patients is summarized in Table 1. Reports on the genetic analysis in the nine patients described previously reported heterozygous MFM‐causing *FLNC* (*n* = 5; P1–4, p.(V930_T933del); P5, p.(K2676Pfs*3)), *DES* (*n* = 2; P7–8; p.(R350P)) and *LDB3* (*n* = 2, P9–10; p.(A147T)) mutations [21, 22, 23, 24, 25]. Patient P6 with a distal myopathy phenotype was reported to harbour a novel heterozygous missense mutation in *FLNC* (c.575G > T; p.(G192V)) leading to an amino acid exchange from glycin to valin in codon 192, which resides in the actin binding domain of the filamin C protein. A predominant proximal pattern of weakness and atrophy was present in six out of nine MFM patients (P1, P3–5, *FLNC* mutation; P9–10; *LDB3* mutation), whereas two MFM patients showed the clinical picture of a distal myopathy (P7–8; *DES* mutation) and a third had a generalized myopathy phenotype (P2; *FLNC* mutation). Slight to moderately elevated CK levels were found in all patients harbouring *DES* and *LDB3* mutation as well as in three out of six individuals with *FLNC* mutations. Serum sodium and potassium levels were within normal limits in all myopathy patients and controls with the exception of patient P10, who had a slightly elevated potassium level (4.9 mmol/L; normal range: 3.6–4.8 mmol/L).

**TABLE 1:** Clinical and genetic data of myofibrillar and distal myopathy patients. An *denote elevated CK levels (reference ranges: < 170/190 U/L in healthy females/males).

Column1	P1	P2	P3	P4	P5	P6	P7	P8	P9	P10
Sex/Age (years)	m/41	m/59	m/48	f/56	f/54	f/53	m/46	m/36	m/57	f/61
Age at onset/Disease duration (years)	39/2	35/24	24/24	30/26	40/14	31/22	34/12	32/4	43/14	48/13
Clinical Phenotype/CK level [U/L]/Mobility score	pm/645* /2	gm/157/4	pm/566* /4	pm/143/3	pm/989* /2	dm/133/3	dm/1204* /2	dm/320* /1	pm/971* /3	pm/218* /3
Mutation/Mode of inheritance	*FLNC* (c.2789_2800del; p.V930_T933del) / sporadic	*FLNC* (c.2789_2800del; p.V930_T933del) / ad	*FLNC* (c.2789_2800del; p.V930_T933del) /ad	*FLNC* (c.2789_2800del; p.V930_T933del) / ad	*FLNC* (c.8025_8030delinsA; p.K2676Pfs*3) /ad	*FLNC* (c.575G > T; p.G192V) /ad	*DES* (c.1049G > C; p.R350P) / ad	*DES* (c.1049G > C; p.R350P) / ad	*LDB3* (c.439G > A; p.A147T) / ad	*LDB3* (c.439G > A; p.A147T) / ad
Reference	Shatunov et al. 2009 [21]	Shatunov et al. 2009 [21]	Shatunov et al. 2009 [21]	Shatunov et al. 2009 [21]	Sellung et al. [22]	unpublished	Bär et al. 2005 [23]	Bär et al. 2005 [23]	Selcen et al. 2005 [24]	Selcen et al. 2005 [24]

MRC‐Grade right/left	P1	P2	P3	P4	P5	P6	P7	P8	P9	P10
Shoulder abduction	5/5	4/4	4/4	2/2	5/5	4+/4+	5/5	5/5	5/5	5/5
Shoulder adduction	5/5	5/5	4/4	5/5	5/5	5/5	5/5	5/5	5/5	5/5
Forearm flexion	5/5	4−/4−	4+/4+	4/4−	4/4	4/4	5/5	4+/4+	4+/4+	4/4
Forearm extension	5/5	4/4	4+/4+	4/4+	5/5	4+/4+	5/5	5/5	5/5	5/5
Wrist flexion	5/5	4/4	5/5	5/5	5/5	4+/4+	5/5	5/5	5/5	4/4
Wrist extension	5/5	5/5	5/5	5/5	5/5	5/5	5/5	5/5	5/5	5/5
Hip flexion	4/4	2/2	1/1	1/1	4−/4−	5/5	4/4	4/4	4/4	4/4
Hip extension	4/4	n.a.	n.a.	4−/4−	5/5	5/5	5/5	5/5	5/5	5/5
Knee flexion	4/4	1–2/1–2	1/1	1/1	4−/4−	4+/4+	4/4	4/4	4/4	3/4
Knee extension	4/4	1–2/1–2	1/1	5/5	4+/4+	4/4	5/5	4/4	4/4	4/4
Foot extension	4−/4—	3/4	3/4—	2–3	3/3	3–4/3–4	4/4	3/3	3/3	3/3
Foot flexion	4−/4−	3/3	4/4	4/4	5/5	3–4/3–4	4/4	4/4	4/4	3/3
Atrophy	Thighs	Shoulders thighs pseudohypertrophy calves		Shoulders thenars thighs	None	Thenar hypothenar calves (medial)		Pectoral finger flexors thenars calves	Pectoral	Thenars

*Note:* Mobility Score: 0 = normal walking distance, 1 = walking distance > 500 m and < 2000 m, 2 = walking distance > 100 m and < 500 m, 3 = walking distance < 100 m, 4 = loss of ambulation.

Abbreviations: CK = creatine kinase; dm = distal myopathy; f = female; gm = generalized myopathy; m = male; MRC = Medical Research Council scale for muscle strength; pm = proximal myopathy.

### 3.2 MRI Findings

#### 3.2.1 T1/T2 STIR—Visual Classification of Fat Replacement/Edema‐Like Alterations

**FIGURE 1:**
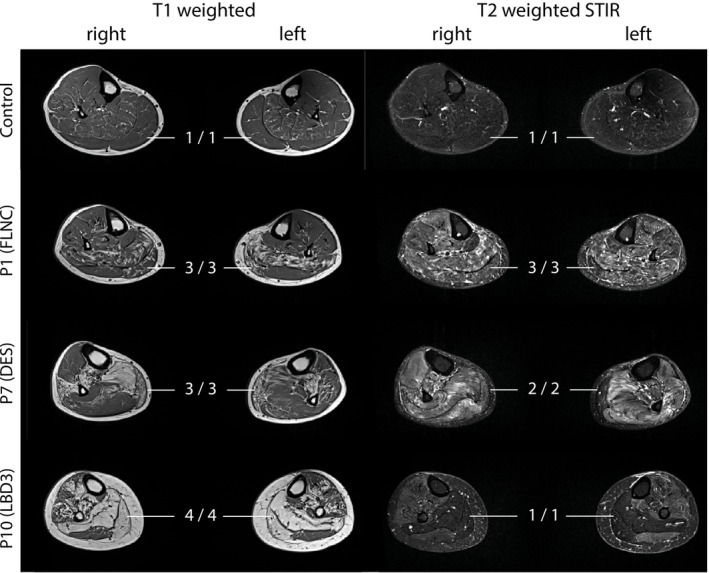
Visual differences in fatty muscle replacement and edema‐like alterations in myofibrillar myopathy patients (MFM). Note the marked differences in the degree of fatty muscle replacement and edema‐like changes between individual muscle regions in the three MFM patients in the T_1_‐weigthed and T_2_‐weigthed STIR ^1^H images of the lower legs. Semi‐quantitative scores for fatty muscle replacement and edema based on the visual assessment of images are given for the medial part of the gastrocnemius muscles. Different muscle compartments are marked in Figure S1 of a control.

To enable visual assessment of fatty muscle replacement and edema‐like alterations, conventional T_1_‐weighted and T_2_‐weighted STIR imaging techniques were applied, respectively. Figure 1 shows representative ^1^H MR images of three patients with different mutation types and one healthy control. Semi‐quantitative scores for fat replacement and edema are summarized in Table 2. In general, there was considerable variability between subjects and between muscles in terms of fat replacement and severity of edema, while lateral differences were minimal. In all patients, regardless of the underlying mutation, less fat‐replaced muscles showed a higher degree of edematous‐like changes. In the five MFM patients who had an *FLNC* mutation, the soleus (SOL) and gastrocnemius medialis (GM) muscles were the most severely replaced by fat, while the peroneus (PER) muscles showed the lowest degree of fat replacement and edema. Patient P6 with *FLNC*‐related distal myopathy also showed the most severe involvement of the GM and SOL muscles, but here the gastrocnemius lateralis (GL) and PER muscles were at least equally affected on one side (Figure S2). The two MFM patients with heterozygous R350P‐DES mutations exhibited an inhomogeneous pattern, with one showing pronounced fatty replacement of the PER, parts of the SOL and GM muscle regions, while the other showed a patchier pattern of fatty replacement affecting all muscle regions with emphasis in the GM and SOL muscles. The two patients with an LBD3 mutation had the highest fat replacement in the GM and SOL muscles and the lowest in the GL muscles.

**TABLE 2:** Semi‐quantitative scores for fatty muscle replacement and edema‐like changes determined by ^1^H T_1_w and T_2_w STIR images, respectively.

		P1	P2	P3	P4	P5	P6	P7	P8	P9	P10
T_1_w	GM	3/3	4/4	4/4	4/4	4/4	4/4	3/3	4/4	4/4	4/4
	GL	2/2	4/4	3/3	4/3	2/2	4/3	1/2	3/4	2/2	2/2
	SOL	3/3	4/4	4/4	4/4	3/3	4/4	3/2	3/3	4/4	4/4
	TA	2/2	4/4	3/3	4/4	4/4	2/2	1/2	3/3	3/3	3/3
	TP	2/2	4/4	3/3	4/4	3/3	2/2	3/3	3/3	2/2	3/4
	PER	1/1	3/3	3/3	4/4	2/2	3/4	3/2	3/3	4/3	3/3
	EDL	1/1	3/3	3/3	4/4	4/4	3/3	2/2	3/3	3/3	3/3
T_2_w STIR	GM	3/3	1/1	1/1	1/1	1/1	1/1	2/2	1/1	1/1	1/1
	GL	3/3	1/1	3/3	3/1	2/3	1/1	4/4	4/1	1/1	4/4
	SOL	4/4	1/1	1/1	1/1	3/3	2/2	4/4	3/3	4/4	1/1
	TA	4/3	2/1	2/2	2/2	3/3	4/4	4/4	4/4	4/4	3/3
	TP	4/4	1/1	2/2	3/3	2/2	4/4	4/4	4/4	3/3	2/2
	PER	2/2	1/1	2/2	2/2	2/2	3/2	3/4	4/4	2/2	4/4
	EDL	2/2	2/2	2/2	2/2	3/3	4/4	4/4	4/4	4/4	4/4

*Note:* The semi‐quantitative scoring (1 = no fat replacement/edema; 2 = slight fat replacement/edema‐like changes, 3 = marked fat replacement/edema‐like changes; 4 = complete fat replacement/edema‐like changes of entire muscle) of right/left lower legs is based on visual assessment. Scoring values are additionally shaded from light to dark grey.

Abbreviations: Extensor digitorum longus (EDL); lateral part of gastrocnemius (GL); medial part of gastrocnemius (GM); peroneal (PER); soleus (SOL); tibialis anterior (TA); tibialis posterior (TP) muscles.

#### 3.2.2 Proton‐Density Fat‐Fraction (PDFF) /Water T_2_ — Quantification of Fat Replacement and Edema‐Like Alterations

**FIGURE 2:**
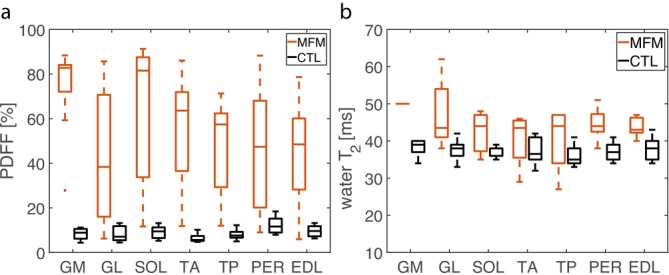
Quantifiable increase of fat replacement and edema‐like alterations in myofibrillar myopathy patients (MFM). Note that proton‐density fat fraction (PDFF) and water T_2_ were higher in MFM patients in all muscular compartments but tibialis anterior and tibialis posterior muscles for water T_2_, demonstrating a quantifiable increase of fat replacement and edema‐like alterations. Boxplots illustrate the results of quantitative assessment of (a) fatty replacement by PDFF and (b) edema‐like alterations by water T_2_, in lower leg muscle regions of nine MFM patients and 10 healthy controls (CTL). For evaluation of water T_2_ only muscles with a mean PDFF < 50% were considered. Please note that water T_2_ evaluation of the medial part of gastrocnemius (GM) muscle is only based on a single patient and thus should be interpreted with caution. Medial part of gastrocnemius (GM); lateral part of gastrocnemius (GL); soleus (SOL); tibialis anterior (TA); tibialis posterior (TP), peroneal (PER); extensor digitorum longus (EDL) muscles.

As quantitative markers for muscular fat content and edema‐like change, PDFF and water T_2_ values were quantified, respectively. For each subject, these quantitative MRI measures were determined in seven individual lower leg muscle regions from one leg, resulting in a total number of 140 evaluated muscles (70 for each the patient and control groups). Figure 2 shows boxplots of the resulting quantitative MRI measures for MFM patients compared to controls. Muscular PDFF values were significantly increased in MFM patients compared to controls in all lower leg muscle regions (*p =* 0.003 to < 0.001, *r*_eff_ = 0.66–0.83, see Figure 2a, heatmaps for individual muscles see Figure S3a). Overall, 60/63 muscles of MFM patients showed an elevated mean PDFF (> 10%) and 35/63 muscles were highly fatty replaced (PDFF > 50%). The median water T_2_ was significantly increased in all muscle regions except for TA and TP muscles of MFM patients compared to controls (*p* = 0.03 to < 0.001, *r*_eff_ = 0.63–0.88, see Figure 2b, heatmaps for individual muscles see Figure S3b). However, for the GM muscle, only one muscle of an MFM patient with *FLNC* mutation could be included due to the PDFF restriction. Median water T_2_ correlated weakly with PDFF when considering all muscular regions in all MFM patients and controls (*r* = 0.34, *p* < 0.001, see Figure 5a). Patient P6 with DM phenotype presented with an elevated mean PDFF (10%–50%) in TA and TP muscle regions, whereas all other muscles were heavily fatty replaced (PDFF > 50%). Boxplots comparing the different genotypes are presented in Figure S4, but should be interpreted with caution given the low number of patients per genotype.

#### 3.2.3 Apparent Tissue Sodium/Potassium Concentration (aTSC/aTPC) — Ion Homeostasis Quantification

**FIGURE 3:**
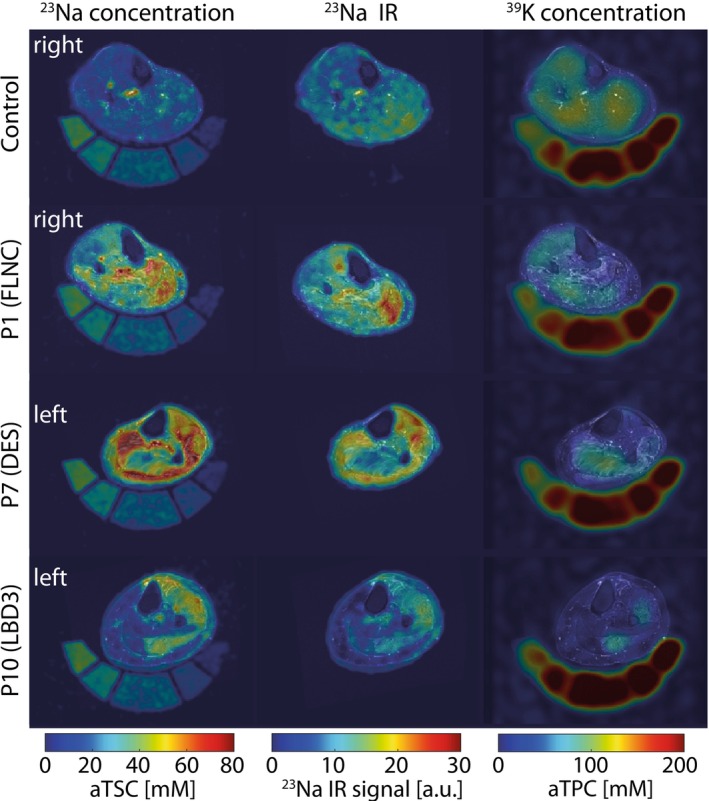
Maps of lower legs of myofibrillar patients (MFM) demonstrating different degrees of increase of apparent tissue sodium concentration (aTSC) and ^23^Na IR signal in non‐complete fatty replaced muscles and decrease of apparent tissue potassium concentration (aTPC) in all muscular regions. Note the increased aTSC/^23^Na IR signal and reduced aTPC in various muscle regions of three myofibrillar myopathy as well as the markedly reduced aTSC and ^23^Na IR signals in the medial part of the gastrocnemius and the soleus muscles of P10, which displayed a high degree of fatty replacement in T_1_ weighted images. ^23^Na and ^39^K MR images are overlaid to corresponding ^1^H T_2_‐weighted STIR images. The same healthy control subject and myofibrillar myopathy patients as in Figure 1 are shown.

To enable a comprehensive analysis of tissue ion homeostasis, sodium and potassium MRI were performed. The obtained aTSC, ^23^Na IR and aTPC maps for the same subjects as in Figure 1 are shown in Figure 3. aTSC was increased in not completely fatty‐replaced muscles and aTPC values were overall reduced in muscles of patients compared to controls. ^23^Na IR signal intensities showed a similar behaviour as aTSC.

**FIGURE 4:**
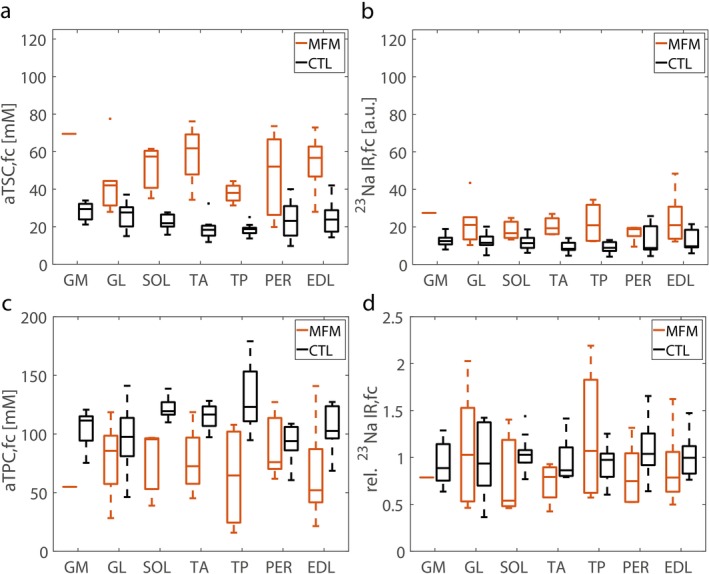
Myofibrillar patients (MFM) show increased apparent tissue sodium concentrations (aTSC), ^23^Na‐Inversion recovery (^23^Na IR) signal intensities and decreased apparent tissue potassium concentrations (aTPC) compared to healthy controls (CTL). ^23^Na IR signal intensities relative to aTSC, showed no difference between the two groups. Quantitative assessment of ion homeostasis by (a) aTSC, (b) ^23^Na IR and (c) aTPC. Relative ^23^Na IR signal intensities with respect to aTSC are presented in (d), hereby all values that conform a linear regression between aTSC and ^23^Na IR signal intensity determined in all muscle regions of all controls were set to 1. Values were fat‐corrected (fc) with the help of mean proton‐density fat‐fractions (PDFF). Only muscles with a mean PDFF < 50% were considered. Please note that evaluation of the medial part of gastrocnemius (GM) muscle is only based on one single muscle and thus should be interpreted with caution. Medial part of gastrocnemius (GM); lateral part of gastrocnemius (GL); soleus (SOL); tibialis anterior (TA); tibialis posterior (TP), peroneal (PER); extensor digitorum longus (EDL) muscles.

Fat‐corrected aTSC values were significantly increased in all seven muscle regions of all MFM patients compared to healthy controls except the PER muscles (*p* = 0.008 to < 0.001, PER 0.059, see Figure 4a, heatmaps for individual muscles see Figure S5a) and accordingly increased when the mean value is calculated across all muscle compartments (58.5 mM, 55.6 ± 16.3 mM vs. 24.3 mM, 23.2 ± 5.5 mM, *p* < 0.001, *r*_eff_ = 0.81). Fat‐corrected ^23^Na IR signal intensities were increased in all muscle regions except for the PER region (*p* = 0.02–0.001; PER *p* = 0.23, see Figure 4b, heatmaps for individual muscles see Figure S5b) with consequent increase of the mean value across all muscle regions (24.8 a.u., 32.5 ± 19.4 a.u. vs. 10.3 a.u., 11.7 ± 4.2 a.u., *p* < 0.001, *r*_eff_ = 0.76). However, when calculating relative fat‐corrected ^23^Na IR signal intensities with respect to aTSC to better assess the impact of MFM on the intracellular weighting of the ^23^Na signal, no significant difference was observed for the MFM patients across all muscular compartments, nor in the mean value (0.98, 1.22 ± 0.63 vs. 0.95, 1.00 ± 0.23, *p* = 0.84, see Figure 4d, heatmaps for individual muscles see Figure S5d). When examining individual muscles of MFM patients, the majority fell within the lower control range as aTSC increased, resulting in a negative correlation between relative ^23^Na IR SI and aTSC (*r* = −0.69, *p* = 0.005). However, two muscles (GL and TP) of patient P9 with an LBD3 mutation showed particularly high values (rel. ^23^Na IR SI > 2, see Figure 6b).

**FIGURE 5:**
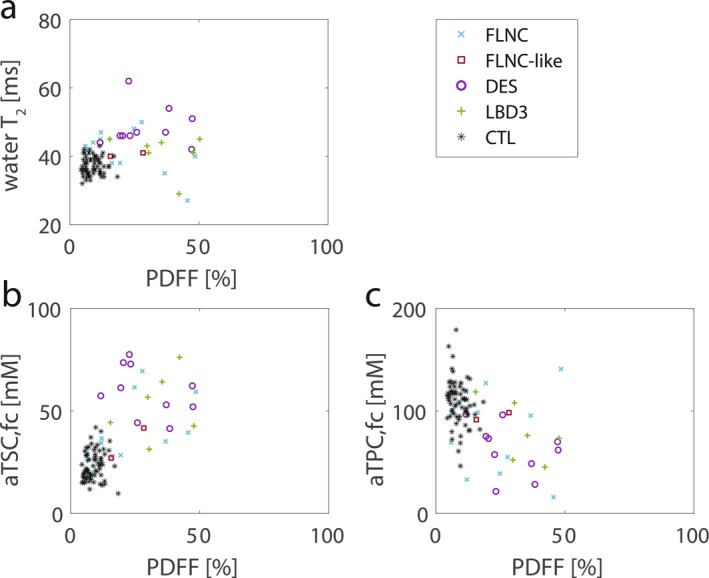
Correlations between quantitative MRI measures at 3 T and 7 T of water T_2_ values, fat‐corrected (fc) apparent tissue sodium and potassium concentrations (aTSC/aTPC) and proton‐density fat fraction (PDFF). (a) Correlation analysis of water T_2_ and PDFF showed a weak correlation of water T_2_ and PDFF for MFM patients for PDFF < 50% (*r* = 0.34, *p* < 0.001), demonstrating that slight to moderate fatty‐replaced muscles also showed an increase in edema‐like alterations. (b) Correlation analysis of the dependence of aTSC on PDFF showed that individual muscles with a low PDFF showed lower aTSC values in the control range, while others already had increased aTSC values. Muscles with increased PDFF values, in turn, usually showed increased aTSC values in the remaining muscle. Correlation between aTSC and PDFF was insignificant for MFM patients (*r* = 0.37, *p* = 0.06) for PDFF < 50%. (c) aTPC showed a similar, but inverse behaviour to aTSC in low PDFF ranges, but at higher PDFF values there was a stronger decrease in aTPC with an increase in PDFF (*r* = −0.46, *p* = 0.02). Note each symbol represents a single muscle or muscle region of a patient with MFM (FLNC, DES or LBD3 mutation), patient P6 with distal myopathy (FLNC‐like mutation) or a control (CTL).

**FIGURE 6:**
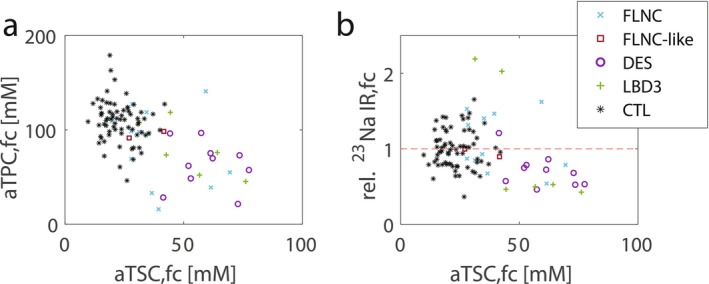
Correlations between apparent tissue potassium concentration (aTPC), relative ^23^Na IR signal intensities and apparent tissue sodium concentration (aTSC). (a) Fat‐corrected (fc) ion homeostasis of K^+^ represented by aTPC correlated moderately negatively with that of Na^+^ expressed by aTSC (*r* = −0.51, *p* < 0.001). (b) Relative ^23^Na IR signal intensities were calculated with respect to aTSC, hereby all values that conform to a linear regression between aTSC and ^23^Na IR signal intensity determined from all muscle regions in all controls were set to 1 (represented by a dashed red line in the plot). The majority of MFM muscles fell within the lower control range, especially for higher aTSC values, resulting in a negative correlation between relative ^23^Na IR signal intensity and aTSC (*r* = −0.69, *p* = 0.005), indicating that changes were more driven by an increase in extracellular volume fraction. Note each symbol represents a single muscle or muscle region of a patient with MFM (FLNC, DES or LBD3 mutation), patient P6 with distal myopathy (FLNC‐like mutation) or a control (CTL).

In contrast to aTSC and ^23^Na IR signal intensities, fat corrected aTPC values were reduced in all muscles but GL, TP and PER (*p* = 0.03 to < 0.001, GL/PER 0.99–0.13; see Figure 4c, heatmaps for individual muscles see Figure S5c) and displayed a decrease of the mean value (76.9 mM, 75.4 ± 13.3 mM vs. 106.0 mM, 108.9 ± 9.9 mM, *p* < 0.001, *r*_eff_ = 0.81). In consequence, a moderate negative correlation between fat‐corrected aTPC and aTSC was observed for MFM patients (*r* = −0.41, *p* = 0.03, see Figure 6a). The correlation analysis of the dependence of aTSC on PDFF showed that individual muscles with a low PDFF showed lower aTSC values in the range of healthy controls, while others already had increased aTSC values (see Figure 5b). Muscles with increased PDFF values, in turn, usually showed increased aTSC values in the remaining muscle. The correlation between aTSC and PDFF was therefore not significant for the MFM patients (*r* = 0.37, *p* = 0.06) for PDFF < 50%. aTPC showed a similar behaviour to aTSC in low PDFF ranges with opposite sign, but at higher PDFF values there was a stronger decrease in aTPC with an increase in PDFF (*r* = −0.46, *p* = 0.02, see Figure 5c).

In patient P6, only two muscles, namely TA and TP (PDFF < 50%), could be evaluated. TA muscle showed a similar pattern as the group of MFM patients with elevated fat‐corrected aTSC and ^23^Na IR SI (aTSC = 41.7 mM, ^23^Na IR = 18.7 a.u.). In contrast, aTPC was decreased (aTPC = 98.3 mM, see also Figure S2). TP muscle of patient P6 showed aTSC and ^23^Na IR SI within the range of controls and a decrease of aTPC (aTSC = 27.0 mM, ^23^Na IR = 13.6 a.u. and aTPC = 91.4 mM). Boxplots comparing the different genotypes are presented in Figure S6, but should be interpreted with caution given the low number of patients per genotype.

## 4 Discussion

In the present study, we performed a multi‐parametric MRI approach to assess the structural and functional skeletal muscle pathology in nine myofibrillar myopathy patients and one patient with FLNC‐related distal myopathy. All nine former patients harboured previously reported heterozygous myofibrillar myopathy‐causing *FLNC*, *DES* or *LDB3* mutations [21, 22, 23, 24, 25], whereas the genetic analysis in patient P6 with a distal myopathy phenotype disclosed a novel heterozygous missense mutation in *FLNC* (c.575G > T; p.G192V) residing in the actin binding domain of the filamin C protein. The findings in the here reported patient strongly mirror the results of a study on an Italian family with distal myopathy due to a heterozygous missense mutation in *FLNC* (c.577G > A; p.A193T), affecting codon 193 [26].

### 4.1 From the Visual Classification to a Reader‐Independent Quantification of Fat Replacement/Edema‐Like Alterations in MFM Muscles

Visual assessment of fatty muscular replacement by semi‐quantitative scoring of T_1_‐weigthed MRI images currently represents the standard of care in neuromuscular disease to search for specific patterns of involvement and disease progression [5, 10, 27].

The patient with non‐MFM, FLNC‐related distal myopathy showed a pattern of muscle involvement with a strong fat replacement in GM and SOL muscle regions, as seen in FLNC‐related MFM patients. However, in this patient, a strong fat replacement in the GL and PER muscles was also noted. Analysis of our two LDB3 patients showed the strongest involvement of GM and SOL muscles. GL muscles in these two siblings were least affected. Our results of T_1_‐weighted MRI basically mirror the previously reported pattern of skeletal muscle involvement in the lower legs in MFM patients due to FLNC and DES mutations [5].

As an extended approach to the visual evaluation of the T_1_‐weighted images, we applied quantitative assessment of PDFF. PDFF determination in the here analysed patients showed in almost all muscles a moderate fatty replacement (PDFF > 10%) and in just over half of the muscles examined a severe fatty replacement (PDFF > 50%). In contrast to the analysis of T_1_‐weighted images by a radiologist, this method enables a reliable, reader‐independent assessment of this disease feature, which could be particularly useful in follow‐up studies and future clinical trials when defining imaging timepoints, as it depicts slight changes in PDFF [28]. These points are particularly valuable in clinical studies for novel therapies. At last, fat correction of aTSC and aTPC, as in this study, is only possible with numerical PDFF quantification.

A second goal of this study was to assess edema‐like changes in our patient cohort. In the clinical setting, this is usually done by evaluating T_2_‐weighted STIR images [6, 27]. In our cohort of patients, edema‐like changes were more pronounced in muscles which only showed mild to moderate fatty replacement than in those with severe fatty replacement.

In addition, we determined muscular water T_2_ values, which allow us to quantify this in a more precise and reader‐independent manner. Our study demonstrated that muscular water T_2_ values were increased in all muscle regions of MFM patients, with the exception of TA and TP muscles. Increases in this parameter, which is believed to indicate early stages of the disease due to edema, inflammation, myocyte swelling or necrosis, have already been reported for several other neuromuscular diseases, such as in the thenar muscles in Duchenne muscular dystrophy (DMD), where it has been suggested as a follow‐up parameter for clinical studies [29]. In principle, the advantages of determining water T_2_ compared to visual semi‐quantitative assessment are the same as in the case of assessment of T_1_‐weighted images compared to PDFF quantification. However, as the applied fit approach is no longer reliable for PDFF values > 50%, visual assessment of T_2_‐weighted STIR images still offers advantages in this setting.

### 4.2 MFM Muscles Display Alterations of the ^39^K/^23^Na Ion Balance

In a final step, we assessed the hypothesis that ion homeostasis of K^+^ and Na^+^_,_ which are known to exert an essential role in muscular excitation and contraction, may be altered in MFM.

Irrespective of the heterogeneity of gene defects and disease progression, fat‐corrected aTSC values were increased and fat‐corrected aTPC values were decreased in a majority of muscular compartments within the lower legs of MFM patients. Notably, parts of muscles with a low‐fat replacement, as indicated by low PDFF values, already showed this pattern, whereas some were still in the range of controls. In general, fat‐corrected aTPC decrease correlated with PDFF increase.

These results indicate that combined aTSC and aTPC measurements may depict changes in the Na^+^/K^+^ homeostasis even in diseased muscles that have not yet undergone advanced fat replacement in MFM patients (PDFF > 50%). It is noteworthy that similar trends for aTSC and aTPC were observed in patient P6 within FLNC‐related distal myopathy, who served as an FLNC, non‐MFM disease control. Thus, aTSC and aTPC may not differentiate between these entities.

While previous studies demonstrated an increase of aTSC in FSHD, DMD and hypo‐ and hyperkalemic periodic paralysis [11, 19, 30, 31], only very limited systematic data are available for aTPC imaging in normal [16, 32, 33] and diseased human skeletal muscle tissue [34].

The reason for the here observed changes in the Na^+^/K^+^ ion homeostasis in MFM patients is currently unclear. In principle, those changes can either be attributed to a change in the intra‐ and extracellular ion concentrations or to an increase in the extracellular volume fraction. To better assess this issue, relative ^23^Na signal intensities were calculated with reference to aTSC. A value of 1 here indicates that the ratio between ^23^Na IR and aTSC corresponds to the mean of the controls. Values > 1 indicate a higher intracellular sodium fraction, whereas values < 1 suggest an increase in extracellular volume. The fact that ^23^Na IR signal intensities relative to aTSC were within the control range (except for two muscles in a patient with an LDB3 mutation) suggests that, in most cases, the observed changes are driven by a combination of changes in Na^+^/K^+^ ion concentrations and an increase in the extracellular volume fraction, assuming that T_1_‐relaxation times remain relatively constant. The fact that the relative ^23^Na IR signal intensities for muscles with higher PDFFs tend to be in the lower control range could indicate that, as muscle alterations progress, the increase in extracellular volume becomes the more dominant factor.

In order to explain the changes in aTSC or aTPC solely with a change in the extracellular volume, there would have to be an increase in the extracellular volume fraction from 0.07 described for healthy controls [35] to 0.32 for aTSC and to 0.35 for aTPC. In this approach, a constant intra‐ and extracellular ion concentration between MFM patients and controls was assumed, with the extracellular concentrations set to the mean serum levels of patients and controls, respectively.

The reason for the observed actual changes of intra‐ and extracellular concentration changes of Na^+^/K^+^ is an unresolved issue as the role of ion homeostasis in skeletal muscle of MFM patients has not yet been investigated. However, in another type of muscular dystrophy, DMD, elevated sodium concentrations have been demonstrated by microelectrode measurements and in muscle biopsies in patients and mice (summarized here: [36]). In DMD, the elevation of Na^+^ is thought to be associated with sarcolemmal instability and dysfunction of ion channels and/or the Na^+^/K^+^ pump. Notably, iPSC‐derived patient‐specific cardiomyocytes of patients with FLNC mutations have recently been described to show changes in calcium dynamics and Na_v_1.5 channels [37]. Corresponding studies are needed to assess changes in Na^+^/K^+^ in skeletal muscle of MFM patients.

Finally, it should be noted that in other muscle diseases such as DMD, mechanisms such as the pump‐leak/Donnan mechanism exist, which mean that even severe changes in the ion balance lead to cytotoxic cell swelling but not to definitive cell death over longer time frames [36, 38]. In our study, aTSC changes were already measurable in at least some of the less fatty muscle replacements of the MFM patients. Whether the Na^+^/K^+^ changes we demonstrated could actually indicate earlier changes that are potentially (partially) reversible with novel therapies needs to be verified in future longitudinal studies.

### 4.3 Clinical Implications/Outlook

If longitudinal studies with a larger number of MFM patients were to show Na^+^/K^+^ changes in earlier disease stages, the detectability of Na^+^/K^+^ homeostasis could open new opportunities for research and diagnostics, facilitating assessment of disease progression at early stages and monitoring of treatment effects. The PDFF and water T_2_ measurements could be valuable in this context due to their higher accuracy compared to semi‐quantitative methods.

Those methods performed at 3 T, including quantitative PDFF and water T_2_ mapping methods, are easier to transfer to a clinical setting than ion measurements at 7 T. With the latter, a ^23^Na measurement with lower resolution/longer measurement time would also be possible at 3 T with appropriate coil equipment [19]. In general, the implementation of ^23^Na and ^39^K MRI into clinical studies faces significant challenges, including high costs associated with specialized hardware and coils. Both nuclei suffer from inherently low signal‐to‐noise ratio (SNR), which is particularly pronounced for ^39^K due to its lower gyromagnetic ratio. Thus, ^39^K measurements are limited to ultrahigh‐field MR systems (≥ 7 T), restricting the clinical applicability. Additionally, their very short T_2_* relaxation times necessitate the use of specialized acquisition schemes such as ultra‐short echo time (UTE) sequences to capture the rapidly decaying signal, which are not readily available on standard MR systems. Ultimately, further studies are needed to validate reproducibility across different sites and scanner types. In our setting high reproducibility of ^39^K/^23^Na measurements has already been successfully demonstrated in healthy volunteers [39].

### 4.4 Limitations

The main limitation of the study was the inclusion of a small number of MFM patients with different genetic subtypes, who were at different stages of the disease, with advanced stages predominating. Accordingly, a more comprehensive statistical analysis remained difficult, particularly with regard to adjustment for multiple tests, as the muscle compartments of a patient or control subject cannot be considered independent of each other, which in turn is usually a prerequisite for this type of post hoc testing. At least in part, we controlled these intermuscular correlations in multiple testing by using the Dubey–Armitage–Parmar procedure [20]. Moreover, since only five out of 10 patients had a diagnostic muscle biopsy in their medical history, we cannot provide sufficient data for a meaningful comparison between histological features with our MRI findings.

Furthermore, exclusion of highly fatty replaced muscles with a PDFF > 50% could potentially result in an underestimation of later disease stages in quantitative parameters other than PDFF. A flow diagram illustrating the exclusion of muscles for the individual genotypes/patients is shown in Figure S7.

Finally, the accuracy of the applied post‐processing workflow for determination of aTSC and aTPC values is limited. The main source of inaccuracies in the quantification process—exceeding the impact of potential inaccuracies in the segmentation—was the non‐rigid image co‐registration, which was required due to the re‐positioning of the subjects between the ^1^H MRI and ^23^Na/^39^K MRI parts performed on different MRI systems. Even though the legs were positioned similarly during both examination parts, slight deformations of the muscles could not always be excluded. Such differences in deformation were only partially compensated by the image co‐registration due to the low resolution of the ^23^Na MRI data, to which the ^1^H data were co‐registered. This affected mostly the GL muscles due to their direct contact with the reference compartment holder. The accuracy of the aTSC/aTPC determination could be increased by acquiring all data sets — ^23^Na/^39^K and ^1^H MRI — on one system within the same patient position.

## 5 Conclusion

In addition to the routinely used visual inspection of T_1_‐ and T_2_‐weighted STIR images, quantitative PDFF measurements and water T_2_ mapping may serve as valuable tools to objectively quantify fat and edema‐like changes in MFM. Changes in potassium/sodium ion balance in the lower leg muscles of MFM patients could serve as new markers to quantify the extent of changes in individual muscle regions. Further longitudinal evaluation is required to validate whether they are sensitive to changes prior to a high degree of fat replacement.

## Funding

Parts of the work were funded by the German Research Foundation (DFG) (project 500888779/RU5534 MR biosignatures at UHF). C.S.M. and T.B. were funded by the DFG—493624887 (Clinician Scientist Programme NOTICE). T.G. was funded by the DFG (project 497320374).

## Ethics Statement

The study was approved by the local institutional ethics committee (No. 254_21B) and has therefore been performed in accordance with the ethical standards defined in the 1964 Declaration of Helsinki and its later amendments. Written informed consent was obtained from all participants prior to their inclusion in the study.

## Conflicts of Interest

L.V.G. is a current employee of Siemens Healthineers. M.U. is a member of the speakers' bureau of Siemens Healthineers. A.M.N. is a member of the speakers' bureau of Siemens Healthineers. C.S.M., C.H., T.G., C.S., M.T., R.H., B.M., F.B.L., J.V.W., S.H., A.D., T.B. and R.S. declare that they have no conflict of interest.

## Supporting information


**Table S1:** Acquisition parameters for ^1^H MRI performed at 3 T and ^23^Na/^39^K MRI performed at 7 T. Acquisition time (T_Acq_), acquisition‐weighted Stack‐of‐Stars (AW‐SOSt), echo time (TE), flash (FL), flip angle (FA), inversion time (TI), multi‐echo spin‐echo (MESE), short‐tau‐inversion‐recovery (STIR), repetition time (TR), turbo‐spin echo (TSE), T_1_‐weighted (T_1w_), T_2_‐weighted (T_2w_).
**Table S2:** Unadjusted/adjusted *p*‐values for muscular‐specific comparisons of quantitative MRI parameters. Unadjusted *p*‐values were calculated by unpaired *t*‐tests in case normality was indicated by the Lilliefors test or by Mann–Whitney *U* tests otherwise. *p*‐values were adjusted for multiple testing for different muscle compartments by the Dubey–Armitage procedure taking into account intermuscular dependence by calculating intermuscular correlations. Apparent tissue sodium (aTSC) and apparent tissue potassium (aTPC) concentration, fat‐corrected (fc), inversion recovery (IR), proton‐density fat fraction (PDFF), relative (rel.). Medial part of gastrocnemius (GM); lateral part of gastrocnemius (GL); soleus (SOL); tibialis anterior (TA); tibialis posterior (TP), peroneal (PER); extensor digitorum longus (EDL) muscles.
**Figure S1:** Exemplary segmentation of muscle regions of the right lower leg of a control. Medial part of gastrocnemius (GM); lateral part of gastrocnemius (GL); soleus (SOL); tibialis anterior (TA); tibialis posterior (TP), peroneal (PER); extensor digitorum longus muscle (EDL) muscles. Additional segmentations of remaining muscle tissue (RM), subcutaneous fat, labelled tibia and fibula (F).
**Figure S2:** MRI findings in patient P6 suffering from FLNC‐related distal myopathy. The upper row represents T_1_‐weighted and T_2_‐weighted STIR ^1^H images of both lower legs. Note the high degree of fatty replacement of soleus and gastrocnemius as well as the left peroneal muscle group. In contrast, the tibialis anterior extensor digitorum longus and tibialis posterior muscles showed only minor fatty replacement and slight edema. Semi‐quantitative scores for fatty muscle replacement and edema based on the visual assessment of images are given for the medial part of the gastrocnemius muscles. The lower row denotes the corresponding quantitative maps of measured apparent tissue sodium concentration (aTSC), ^23^Na IR signal and apparent tissue potassium concentration (aTPC) at 7 T overlaid to corresponding ^1^H T_2_‐weighted STIR images of the left lower leg. Note the increased aTSC/^23^Na IR signal and reduced aTPC in tibialis anterior (TA), extensor digitorum longus (EDL) and tibials posterior (TP) muscles, whereas the dorsal calf muscles with a high degree of fatty replacement displayed markedly reduced aTSC and ^23^Na IR signals. In least replaced tibialis anterior muscle aTSC = 32.1 mM, ^23^Na IR = 14.1 a.u. and aTPC = 70.5 mM and extensor digitorum longus muscle aTSC = 26.4 mM, ^23^Na IR = 13.8 a.u. and aTPC = 29.5 mM.
**Figure S3:** Heatmaps illustrating muscle specific results of MRI measurements at 3 T in lower leg muscle groups of nine myofibrillar myopathy patients (MFM) and one non‐MFM FLNC‐like distal myopathy (DM) patient. Same ordering as in Table 2. Quantitative assessment of (a) fatty replacement by proton‐density fat fraction (PDFF) and (b) edema‐like alterations by water T_2_. For evaluation of water T_2_ only muscles with a mean PDFF < 50% were considered (other muscles marked in white/NaN).
**Figure S4:** Different genotypes of myofibrillar myopathy (MFM) compared to healthy controls (CTL). FLNC‐like denotes patient P6 with FLNC‐like non‐MFM distal myopathy (DM). Muscle regions of all patients with the respective genotype were pooled together. Quantitative assessment of (a) fatty replacement by proton‐density fat fraction (PDFF) and (b) edema‐like alterations by water T_2_. For evaluation of water T_2_ only muscles with a mean PDFF < 50% were considered. The central line of the box plots represents the median, the bottom and top edges indicate the 25th and the 75th percentiles, respectively. The whiskers extend to most extreme data points not regarded as outliers, ‘+’ symbols indicate outliers.
**Figure S5:** Heatmaps illustrating muscle specific results of MRI measurements at 7 T in lower leg muscle groups of nine myofibrillar myopathy patients (MFM) and one non‐MFM FLNC‐like distal myopathy (DM) patient. Same ordering as in Table 2. Quantitative assessment of ion homeostasis by (a) apparent tissue sodium concentration (aTSC), (b) ^23^Na‐Inversion recovery signal intensities (^23^Na IR) and (c) apparent tissue potassium concentration (aTPC). Relative ^23^Na IR signal intensities with respect to aTSC are presented in (d), hereby all values that conform a linear regression between aTSC and ^23^Na IR signal intensity determined in all muscle regions of all controls were set to 1. Values were fat‐corrected (fc) with the help of mean proton‐density fat‐fractions (PDFF). Only muscles with a mean PDFF < 50% were considered (other muscles marked in white/NaN).
**Figure S6:** Boxplots illustrating the results of quantitative MRI measurements at 3 T in lower leg muscles for different genotypes of myofibrillar myopathy (MFM) compared to healthy controls (CTL). FLNC‐like denotes patient P6 with FLNC‐like non‐MFM distal myopathy (DM). Muscle regions of all patients with the respective genotype were pooled together. Quantitative assessment of ion homeostasis by (a) apparent tissue sodium concentration (aTSC), (b) ^23^Na‐Inversion recovery signal intensities (^23^Na IR) and (c) apparent tissue potassium concentration (aTPC). Values were fat‐corrected (fc) with the help of mean proton‐density fat‐fractions (PDFF). Only muscles with a mean PDFF < 50% were considered. The central line of the box plots represents the median, the bottom and top edges indicate the 25th and the 75th percentiles, respectively. The whiskers extend to most extreme data points not regarded as outliers, ‘+’ symbols indicate outliers.
**Figure S7:** Flow diagram illustrating for myofibrillar myopathy patients (MFM) and one patient with non‐MFM FLNC‐like distal myopathy (DM) the exclusion of heavily fat‐replaced muscles with a proton‐density fat fraction (PDFF) > 50% per genotype and consecutive numbers of evaluated muscles for quantitative parameters other than PDFF.
