# Supplementary material for: Multi‐Parametric MRI Approach at 3 T and 7 T for Assessing Skeletal Muscle Pathology in Myofibrillar Myopathies: A Pilot Study
Source: J Cachexia Sarcopenia Muscle. 2026 Mar 19;17(2):e70245. doi: 10.1002/jcsm.70245 (PMC13140330; doi:10.1002/jcsm.70245)
Supplement: Supplementary file 1 — Table S1: Acquisition parameters for 1H MRI performed at 3 T and 23Na/39K MRI performed at 7 T. Acquisition time (TAcq), acquisition‐weighted Stack‐of‐Stars (AW‐SOSt), echo time (TE), flash (FL), flip angle (FA), inversion time (TI), multi‐echo spin‐echo (MESE), short‐tau‐inversion‐recovery (STIR), repetition time (TR), turbo‐spin echo (TSE), T1‐weighted (T1w), T2‐weighted (T2w). Table S2: Unadjusted/adjusted p‐values for muscular‐specific comparisons of quantitative MRI parameters. Unadjusted p‐values were calculated by unpaired t‐tests in case normality was indicated by the Lilliefors test or by Mann–Whitney U tests otherwise. p‐values were adjusted for multiple testing for different muscle compartments by the Dubey–Armitage procedure taking into account intermuscular dependence by calculating intermuscular correlations. Apparent tissue sodium (aTSC) and apparent tissue potassium (aTPC) concentration, fat‐corrected (fc), inversion recovery (IR), proton‐density fat fraction (PDFF), relative (rel.). Medial part of gastrocnemius (GM); lateral part of gastrocnemius (GL); soleus (SOL); tibialis anterior (TA); tibialis posterior (TP), peroneal (PER); extensor digitorum longus (EDL) muscles. Figure S1: Exemplary segmentation of muscle regions of the right lower leg of a control. Medial part of gastrocnemius (GM); lateral part of gastrocnemius (GL); soleus (SOL); tibialis anterior (TA); tibialis posterior (TP), peroneal (PER); extensor digitorum longus muscle (EDL) muscles. Additional segmentations of remaining muscle tissue (RM), subcutaneous fat, labelled tibia and fibula (F). Figure S2: MRI findings in patient P6 suffering from FLNC‐related distal myopathy. The upper row represents T1‐weighted and T2‐weighted STIR 1H images of both lower legs. Note the high degree of fatty replacement of soleus and gastrocnemius as well as the left peroneal muscle group. In contrast, the tibialis anterior extensor digitorum longus and tibialis posterior muscles showed only minor fatty [file JCSM-17-e70245-s001.docx]

**Supplement to Multi-parametric MRI approach at 3T and 7T for assessing skeletal muscle pathology in myofibrillar myopathies: a pilot study**

**Supplementary tables**

**Table S1**: Acquisition parameters for ^1^H MRI performed at 3 T and ^23^Na/^39^K MRI performed at 7 T. Acquisition time (T_Acq_), acquisition-weighted Stack-of-Stars (AW-SOSt), echo time (TE), flash (FL), flip angle (FA), inversion time (TI), multi-echo spin-echo (MESE), short-tau-inversion-recovery (STIR), repetition time (TR), turbo-spin echo (TSE), T_1_-weighted (T_1w_), T_2_-weighted (T_2w_).

| Acquisition | TR  [ms] | TE  [ms] | TI [ms] | Echo spacing [ms] | FA  [°] | Bandwidth [Hz/Px] | Nominal Resolution  [mm^3^] | Slices | T_Acq_ [min:sec] |
| --- | --- | --- | --- | --- | --- | --- | --- | --- | --- |
| 2D T_1w_ TSE | 588 | 9.9 | - | 9.9 | 150 | 252 | 0.8x0.8x3.0 | 90 | 3:19 |
| 2D T_2w_ STIR TSE | 4370 | 75.0 | - | 9.34 | 145 | 360 | 0.8x0.8x3.0 | 26 | 4:06 |
| MESE | 3000 | 9.5-304 (32 echoes) | - | 9.5 | 90-180 | 449 | 1.3x1.3x10 | 7 | 3:29 |
| FL3D Vibe Dixon | 21 | 2.22- 18.22 (6 echoes) | 200 | 3.2 | 6 | 890 | 0.9x0.9x5 | 50 | 1:57 |
|  |  |  |  | Radial Spokes |  |  |  |  |  |
| ^23^Na AW-SOSt | 120 | 0.3 | - | 253 | 90 | 100 | 2.5x2.5x15 | 16 | 8:04 |
| ^23^Na IR AW-SOSt | 120 | 0.3 | 34 | 269 | 180-90 | 100 | 3.5x3.5x15 | 16 | 8:36 |
| ^39^K AW-SOSt | 40 | 0.35 | - | 84 | 90 | 200 | 7.5x7.5x30 | 8 | 8:58 |

**Table S2:** Unadjusted/adjusted p-values for muscular-specific comparisons of quantitative MRI parameters. Unadjusted p-values were calculated by unpaired t-tests in case normality was indicated by the Lilliefors test or by Mann-Whitney-U tests otherwise. P-values were adjusted for multiple testing for different muscle compartments by the Dubey-Armitage procedure taking into account intermuscular dependence by calculating intermuscular correlations. Apparent tissue sodium (aTSC) and apparent tissue potassium (aTPC) concentration, fat-corrected (fc), inversion recovery (IR), proton-density fat fraction (PDFF), relative (rel.). Medial part of gastrocnemius (GM); lateral part of gastrocnemius (GL); soleus (SOL); tibialis anterior (TA); tibialis posterior (TP), peroneal (PER); extensor digitorum longus (EDL) muscles.

|  | PDFF | water T_2_ | aTSC,fc | ^23^Na IR,fc | rel. ^23^Na IR,fc | aTPC,fc |
| --- | --- | --- | --- | --- | --- | --- |
| GM | 2.17E-05  /2.77E-05 | 3.11E-04  /4.58E-04 | 8.28E-04  /2.88E-03 | 1.57E-03  /2.94E-03 | 0.02  /0.02 | 7.58E-03  /0.03 |
| GL | 2.04E-03  /2.60E-03 | 9.43E-03  /0.01 | 2.24E-03  /7.77E-03 | 1.03E-02  /0.02 | 0.43  /0.58 | 0.68  /0.99 |
| SOL | 4.33E-05  /5.53E-05 | 0.02  /0.03 | 2.60E-04  /9.04E-04 | 1.45E-03  /2.71E-03 | 0.25  /0.35 | 1.03E-03  /4.51E-03 |
| TA | 1.73E-05  /2.21E-05 | 0.28  /0.38 | 2.17E-05  /7.53E-05 | 3.99E-03  /7.45E-03 | 0.82  /0.93 | 8.89E-05  /3.89E-04 |
| TP | 1.24E-05  /1.58E-05 | 0.17  /0.24 | 8.22E-05  /2.86E-04 | 7.33E-04  /1.37E-03 | 0.24  /0.34 | 0.03  /0.13 |
| PER | 1.03E-03  /1.31E-03 | 1.05E-03  /1.54E-03 | 0.02  /0.06 | 0.13  /0.23 | 0.47  /0.62 | 0.13  /0.47 |
| EDL | 1.43E-04  /1.82E-04 | 2.36E-03  /3.47E-03 | 1.22E-04  /4.24E-04 | 0.01  /0.02 | 0.88  /0.96 | 5.46E-03  /0.02 |

**Supplementary figures**


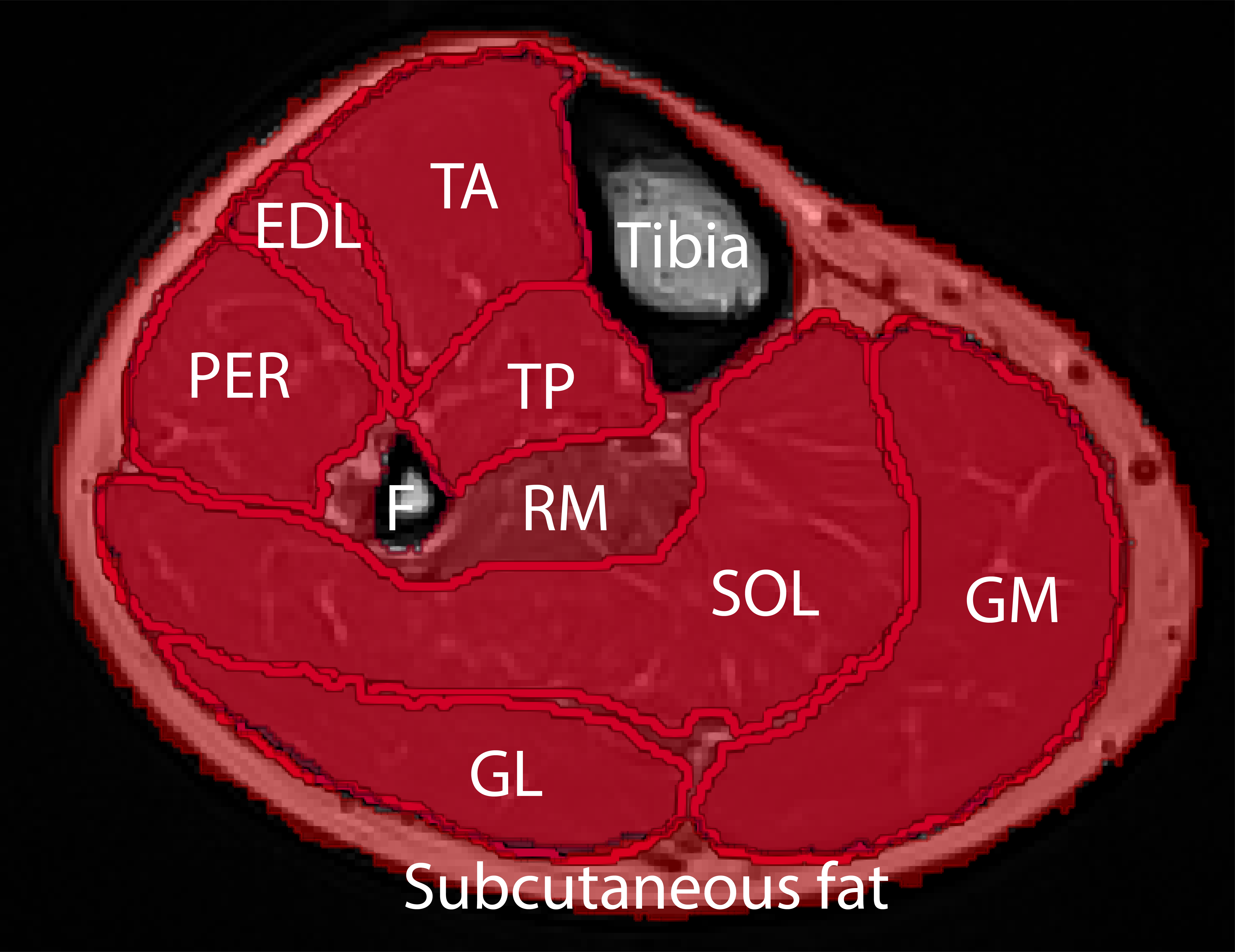


**Figure S1:** Exemplary segmentation of muscle regions of the right lower leg of a control. Medial part of gastrocnemius (GM); lateral part of gastrocnemius (GL); soleus (SOL); tibialis anterior (TA); tibialis posterior (TP), peroneal (PER); extensor digitorum longus (EDL) muscles. Additional segmentations of remaining muscle tissue (RM), subcutaneous fat, labeled tibia and fibula (F).


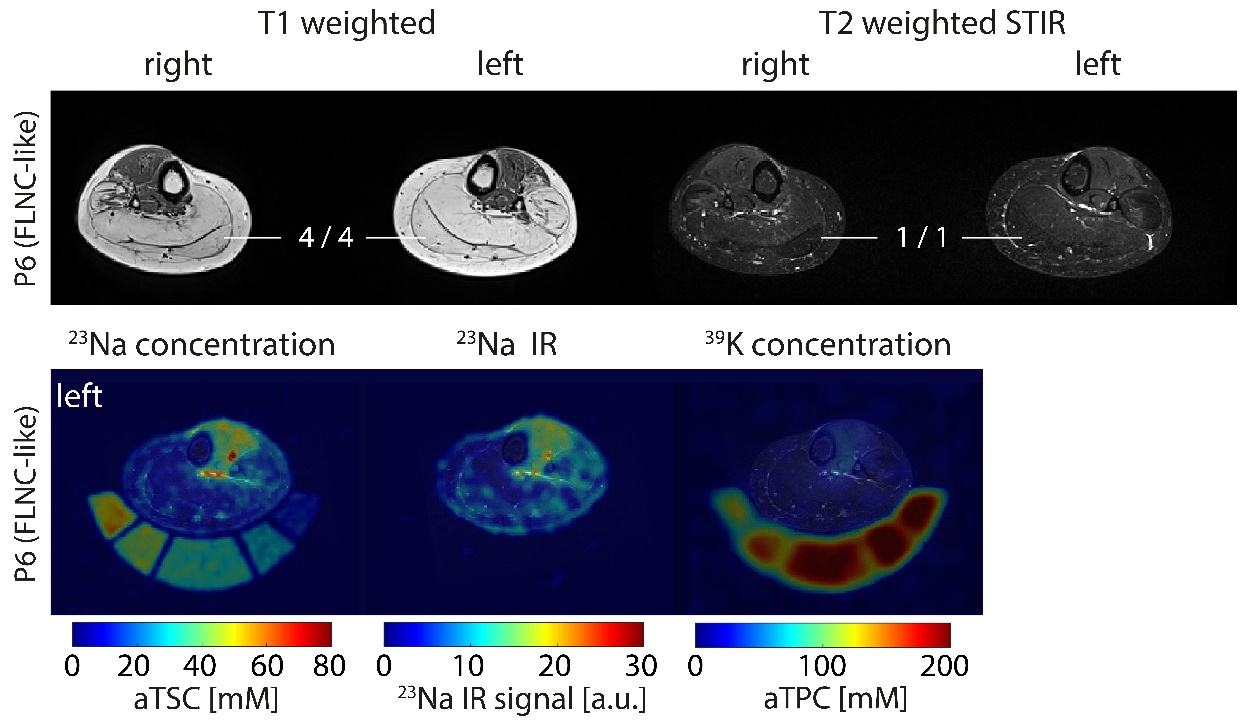


**Figure S2:** MRI findings in patient P6 suffering from FLNC-related distal myopathy. The upper row represents T_1_-weighted and T_2_-weighted STIR ^1^H images of both lower legs. Note the high degree of fatty replacement of soleus and gastrocnemius as well as the left peroneal muscle group. In contrast, the tibialis anterior extensor digitorum longus and tibialis posterior muscles showed only minor fatty replacement and slight edema. Semi-quantitative scores for fatty muscle replacement and edema based on the visual assessment of images are given for the medial part of the gastrocnemius muscles. The lower row denotes the corresponding quantitative maps of measured apparent tissue sodium concentration (aTSC), ^23^Na IR signal and apparent tissue potassium concentration (aTPC) at 7 T overlaid to corresponding ^1^H T_2_-weighted STIR images of the left lower leg. Note the increased aTSC/^23^Na IR signal and reduced aTPC in tibialis anterior (TA), extensor digitorum longus (EDL) and tibials posterior (TP) muscles, whereas the dorsal calf muscles with a high degree of fatty replacement displayed markedly reduced aTSC and ^23^Na IR signals. In least replaced tibialis anterior muscle aTSC = 32.1 mM, ^23^Na IR = 14.1 a.u. and aTPC = 70.5 mM and extensor digitorum longus muscle aTSC = 26.4 mM, ^23^Na IR = 13.8 a.u. and aTPC = 29.5 mM.


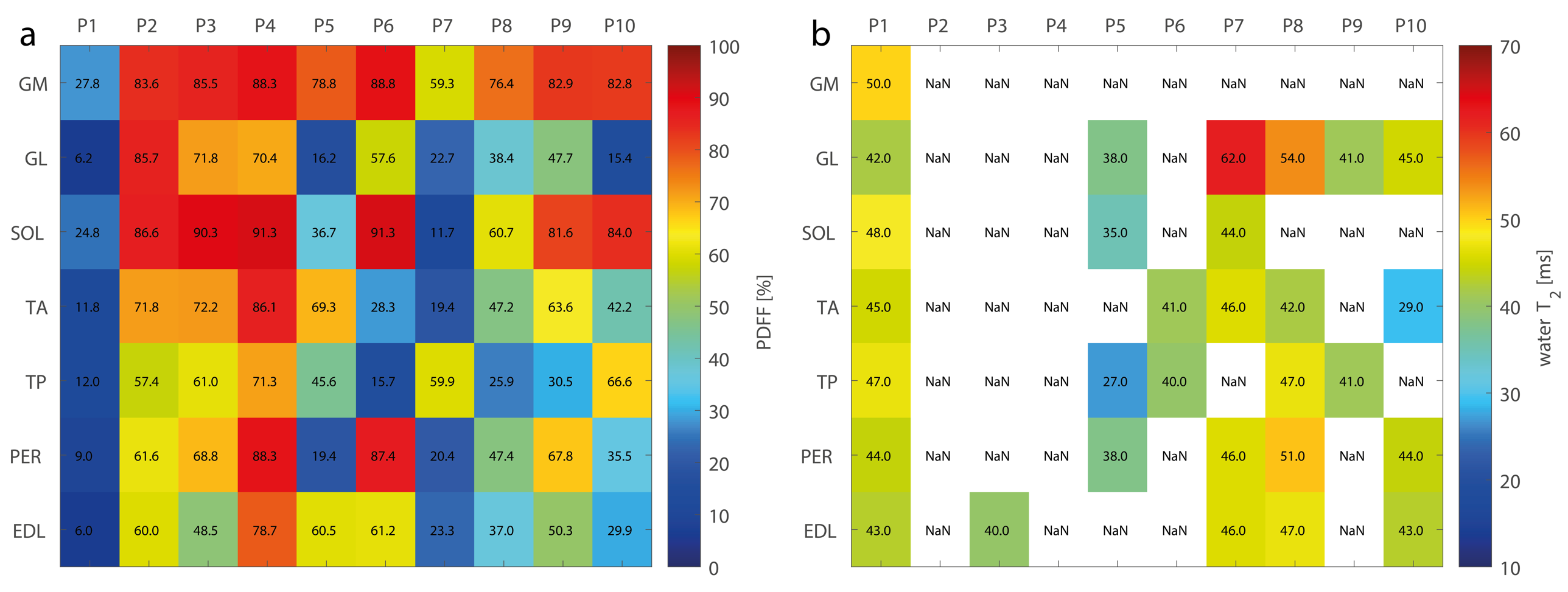


**Figure S3:** Heatmaps illustrating muscle specific results of MRI measurements at 3 T in lower leg muscle groups of nine myofibrillar myopathy patients (MFM) and one non-MFM FLNC-like distal myopathy (DM) patient. Same ordering as in Table 2. Quantitative assessment of a) fatty replacement by proton-density fat fraction (PDFF) and b) edema-like alterations by water T_2_. For evaluation of water T_2_ only muscles with a mean PDFF <50% were considered (other muscles marked in white/NaN).


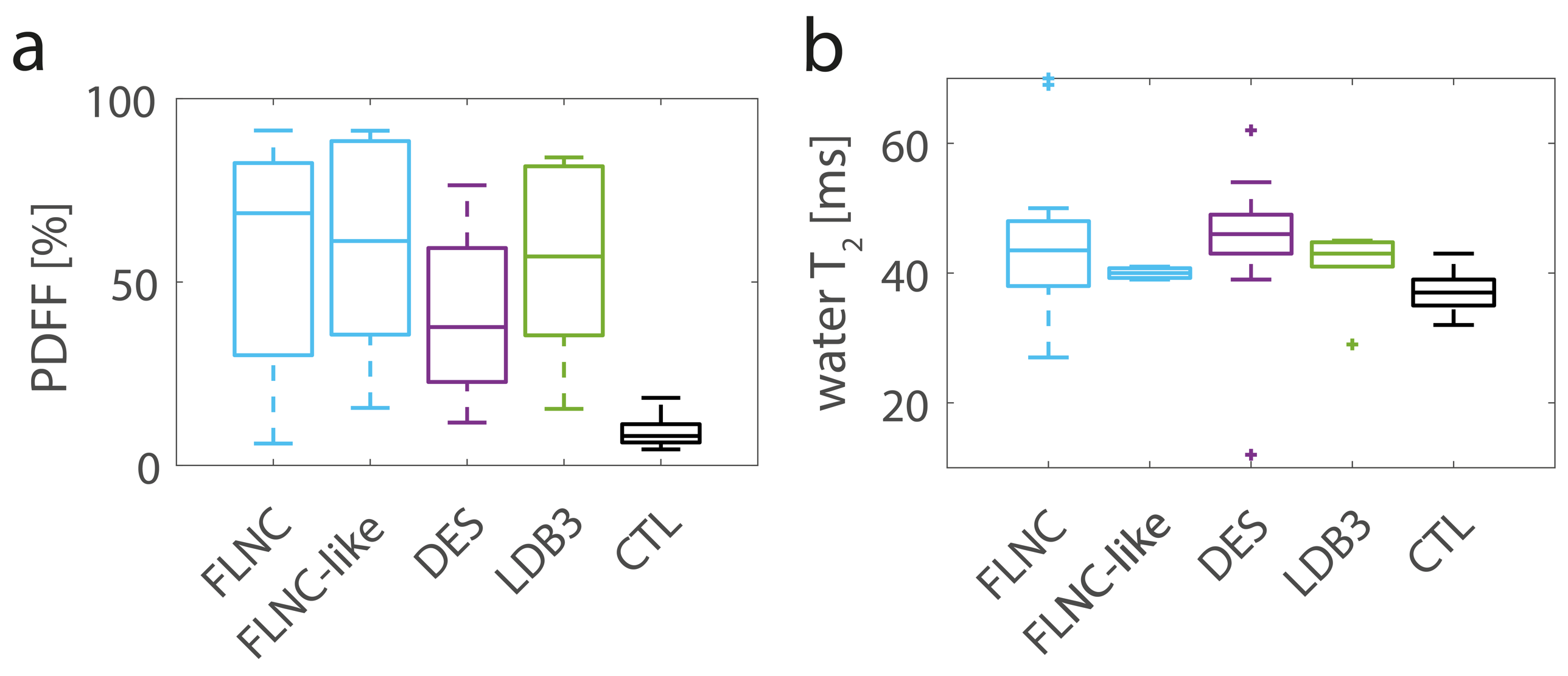


**Figure S4:** Different genotypes of myofibrillar myopathy (MFM) compared to healthy controls (CTL). FLNC-like denotes patient P6 with FLNC-like non-MFM distal myopathy (DM). Muscle regions of all patients with the respective genotype were pooled together. Quantitative assessment of a) fatty replacement by proton-density fat fraction (PDFF) and b) edema-like alterations by water T_2_. For evaluation of water T_2_ only muscles with a mean PDFF <50% were considered. The central line of the box plots represents the median, the bottom and top edges indicate the 25th and the 75th percentiles, respectively. The whiskers extend to most extreme data points not regarded as outliers, ‘+’ symbols indicate outliers.





**Figure S5:** Heatmaps illustrating muscle specific results of MRI measurements at 7 T in lower leg muscle groups of nine myofibrillar myopathy patients (MFM) and one non-MFM FLNC-like distal myopathy (DM) patient. Same ordering as in Table 2. Quantitative assessment of ion homeostasis by a) apparent tissue sodium concentration (aTSC), b) ^23^Na-Inversion recovery signal intensities (^23^Na IR) and c) apparent tissue potassium concentration (aTPC). Relative ^23^Na IR signal intensities with respect to aTSC are presented in d), hereby all values that conform a linear regression between aTSC and ^23^Na IR signal intensity determined in all muscle regions of all controls were set to 1. Values were fat-corrected (fc) with the help of mean proton-density fat-fractions (PDFF). Only muscles with a mean PDFF <50% were considered (other muscles marked in white/NaN).





**Figure S6:** Boxplots illustrating the results of quantitative MRI measurements at 3 T in lower leg muscles for different genotypes of myofibrillar myopathy (MFM) compared to healthy controls (CTL). FLNC-like denotes patient P6 with FLNC-like non-MFM distal myopathy (DM). Muscle regions of all patients with the respective genotype were pooled together. Quantitative assessment of ion homeostasis by a) apparent tissue sodium concentration (aTSC), b) ^23^Na-Inversion recovery signal intensities (^23^Na IR) and c) apparent tissue potassium concentration (aTPC). Values were fat-corrected (fc) with the help of mean proton-density fat-fractions (PDFF). Only muscles with a mean PDFF <50% were considered. The central line of the box plots represents the median, the bottom and top edges indicate the 25th and the 75th percentiles, respectively. The whiskers extend to most extreme data points not regarded as outliers, ‘+’ symbols indicate outliers.





**Figure S7:** Flow diagram illustrating for myofibrillar myopathy patients (MFM) and one patient with non-MFM FLNC-like distal myopathy (DM) the exclusion of heavily fat-replaced muscles with a proton-density fat fraction (PDFF) > 50 % per genotype and consecutive numbers of evaluated muscles for quantitative parameters other than PDFF.
